# Factors Associated with Medical Mistrust Among Sexual and Gender Minority Young Adults

**DOI:** 10.1007/s13178-025-01139-y

**Published:** 2025-06-19

**Authors:** Afiya Sajwani, Sarah W. Whitton, Gregory Swann, Michael E. Newcomb

**Affiliations:** 1 Department of Psychiatry & Behavioral Sciences, Northwestern University Feinberg School of Medicine, Chicago, IL USA; 2 Institute for Sexual and Gender Minority Health and Wellbeing, Northwestern University Feinberg School of Medicine, Chicago, IL USA; 3 Psychology Department, University of Cincinnati, Cincinnati, OH USA; 4 Department of Medical Social Sciences, Northwestern University Feinberg School of Medicine, Chicago, IL USA

**Keywords:** Healthcare access, Sexual minority women, Transgender and non-binary, Minority stress, Victimization, Self-rated health

## Abstract

**Introduction:**

Medical mistrust (MM), distrusting medical professionals and systems due to experiences of marginalization, is related to health disparities. Little is known about MM among young cisgender sexual minority women and transgender and non-binary (TNB) young adults. We examined correlates and compared rates of MM among these groups.

**Methods:**

Data came from a longitudinal cohort study, collected between 2019 and 2020. Participants completed measures of demographics (age, race/ethnicity, and gender identity), MM, victimization, internalized stigma, social support, resilience, STI testing, gender-affirming hormone use, and self-rated physical health. Bivariate correlational analyses tested the strength of associations between predictor variables and MM. Variables significantly correlated with MM were included in multiple regression models. Moderating effects of gender identity were also tested.

**Results:**

Analyses included 410 participants (*M* = 22.4, SD = 3.5, 18–33 years, 27% White, 33% TNB). MM was significantly correlated with victimization, resilience, family support, STI testing and self-rated health. In regression analyses, Black and TNB identities, higher victimization, lower family support, and poor self-rated health were significantly associated with higher MM (*R*^2^ = .147, *F*(9, 400) = 8.85, *p* < 0.001). Additionally, MM decreased as family support increased for cisgender, but not TNB participants (*R*^2^ = .152, *F*(17, 392) = 5.32, *p* < 0.000). Comparing model fit statistics, the main effects model best described our data.

**Conclusions:**

More distal instead of proximal minority stressors and family support over other types of social support were associated with reduced MM.

**Policy Implications:**

Reducing general experiences of victimization, increasing family support, and considering intersectional structural stigma can help address MM among these groups.

Medical mistrust (MM) is defined as the distrust of medical professionals and systems due to direct or vicarious experiences of marginalization (Benkert et al., 2019). MM has been identified as a social determinant of health (Institute of Medicine, 2003) and linked with health disparities among marginalized populations. For example, among Black and Latinx individuals, MM is notably associated with delays in preventive screenings (Adams et al., 2017; Bynum et al., 2012; Kinlock et al., 2017) and low satisfaction with healthcare services (LaVeist et al., 2009; Sheppard et al., 2019; Thompson et al., 2004). There is also a substantial body of literature documenting MM among sexual minority populations related to HIV prevention and care (for a review see: Williamson & Bigman, 2018). Research suggests links between MM and increased HIV risk behaviors (e.g., Bogart & Thorburn, 2005; Bogart et al., 2021), poor adherence to anti-retroviral medication (e.g., Meyers-Pantele et al., 2022) and low uptake of pre-exposure prophylaxis (PrEP; e.g., Cahill et al., 2017).

Few studies have explored MM among gender minority populations and sexual minority women. There is evidence to suggest high MM among transgender and non-binary (TNB) adults (e.g., Arrington-Sanders et al., 2020; Cahill et al., 2020; D’Avanzo et al., 2019; Daniels et al., 2019; Eaton et al., 2017; Sevelius et al., 2016). However, these studies are limited in scope due to their focus on TNB individuals accessing HIV- and PrEP-related care. Further, these HIV-focused studies have too often grouped transfeminine individuals together with cisgender sexual minority men (labeling them all as “men who have sex with men”), and when TNB individuals have been examined separately as a group, they were almost always transfeminine individuals. Among sexual minority women (i.e., lesbian, bisexual and queer women), MM has been examined infrequently and only a few studies have investigated the relationship between MM and healthcare engagement (e.g., Brenick et al., 2017; Brotman et al., 2003; Hart & Bowen, 2009). Finally, most studies examining MM among TNB individuals and cisgender sexual minority women have focused on adults (e.g., Brenick et al., 2017; Brotman et al., 2003; Cahill et al., 2020; D’Avanzo et al., 2019; Eaton et al., 2017; Hart & Bowen, 2009; Sevelius et al., 2016), often excluding developmental considerations and perspectives from young adults. Thus, the goal of this study is to examine correlates of MM in TNB young adults (i.e., transgender men and non-binary young adults designated female at birth) and young cisgender sexual minority women (YCSMW) and compare rates of MM between these groups who are underrepresented in the current literature.

TNB young adults and YCSMW experience significant health disparities. Compared to cisgender peers, TNB young adults experience worse mental health outcomes (Becerra-Culqui et al., 2018; Newcomb et al., 2020; Reisner et al., 2015a, 2015b), greater suicidality (Perez-Brumer et al., 2021), substance use (Day et al., 2017), and cardiovascular problems (Nokoff et al., 2020). YCSMW similarly report high rates of substance use (Kerr et al., 2015) and physical health problems, including breast cancer and asthma (Boehmer et al., 2014; Simoni et al., 2017). The minority stress and resilience model has been widely understood to explain health disparities faced by sexual and gender minority (SGM) populations (e.g., Brooks, 1981; Hatzenbuehler, 2009; Hendricks & Testa, 2012; Meyer, 2003). The model posits that the interplay of internal and external stressors unique to SGM individuals results in poor physical and mental health outcomes. Internal or proximal stressors include identity concealment, internalized stigma, and anticipation of stigma, whereas external or distal stressors include discrimination, victimization, rejection, and non-affirmation. Additionally, the model suggests that resilience factors, such as social support and identity pride, may buffer against the negative impacts of minority stressors.

Little research has applied the minority stress and resilience framework to understand the development of MM among SGM populations, but evidence suggests that these experiences may be linked. TNB young adults and YCSMW, in particular, report experiencing victimization in healthcare settings based on their gender identity and sexual orientation (Brenick et al., 2017; Goldenberg et al., 2021; Kearns et al., 2021). Such experiences of victimization within healthcare settings may lead to increased distrust towards medical systems. In fact, among TNB young adults particularly, experiences of victimization in healthcare settings are associated with delayed medical care (Kcomt et al., 2020) — a consequence highly linked with MM among other minoritized populations (Adams et al., 2017). It is possible that overt experiences of victimization (e.g., in healthcare settings) may translate to or reinforce internalized stigma (Testa et al., 2015), which may further contribute to increased medical mistrust. Even in the absence of experiencing stigma in healthcare settings, a lifetime of encountering minority stressors more broadly may contribute to distrust in numerous institutions, including the medical establishment.

Nevertheless, some SGM young adults experience protective factors, like resilience and social support, in the face of minority stress. These may promote health access and outcomes, and may help to reduce MM. Indeed, research has found that social support buffers against the negative impact of stress on numerous health outcomes among TNB young adults and YCSMW (Dowers et al., 2020; Westwater et al., 2019; Whitton et al., 2018). Taken together, minority stressors (e.g., victimization and internalized stigma) and resilience factors (e.g., social support) may be associated with MM among both TNB young adults and YCSMW. Considering increased health disparities experienced by TNB individuals compared to cisgender counterparts, it is likely that associations between MM and victimization, internalized stigma and social support will differ based on gender identity.

Although there is significant evidence for understanding SGM health disparities using the minority stress and resilience framework (Delozier et al., 2020), this framework likely cannot completely explain the development of MM among these young adults. Upon examining existing literature on MM, we identified additional factors not captured by the minority stress and resilience framework which may contribute to developing or sustaining MM among SGM young adults. Evidence suggests that having positive experiences within the healthcare system is associated with reduced MM (Hall et al., 2001; Lee & Lin, 2008). One such healthcare experience that many SGM young adults regularly access is testing for sexually transmitted infections (STI). STI testing services are unique from other healthcare services in that they are often performed in the context of specialty clinics, many of which may be more SGM-affirming compared to traditional medical settings (Bauermeister et al., 2015, 2019). As such, receiving STI testing may be associated with reduced MM among SGM young adults. However, affirming STI testing services have historically been designed for those groups with the highest number of infections (i.e., cisgender sexual minority men; Stewart et al., 2022), so it is possible that TNB young adults and YCSMW may feel unaffirmed in these environments, which may exacerbate MM. Among TNB young adults, pursuing gender-affirming hormones (GAH) to meet transition-related goals likely reflects a positive medical experience for many, and therefore, accessing GAH may be associated with less MM. Conversely, not receiving GAH when desired may reflect systemic barriers to receiving such care which may reinforce or increase MM. Taken together, it stands to reason that affirming healthcare experiences, in the context of STI testing or pursuing GAH, may be associated with decreased MM.

MM has also been linked to perceptions of one’s health, such that poor perceptions of health are associated with increased MM (Armstrong et al., 2006; Mohseni & Lindstrom, 2007; Yang et al., 2011). Self-perceptions of health are generally understood to be valid indicators of health status (Miilunpalo et al., 1997) and related to various health outcomes (Benyamini, 2011; Idler & Benyamini, 1997). Individuals reporting poor self-rated health may have more frequent interactions with the health system and may perceive these interactions as negative. Research indicates that TNB individuals and cisgender sexual minority women report poor self-rated overall health (Diamond et al., 2021; Gorman et al., 2015; Przedworski et al., 2014). These disparities appear to be particularly pronounced among TNB individuals. For example, a population study of 80,929 high-school students observed that TNB students rated their physical health as significantly worse compared to cisgender peers, even in the absence of a reported health condition (Rider et al., 2018). In addition to poor self-rated health, TNB young adults and YCSMW report underutilizing health services (Charlton et al., 2011; Pharr et al., 2019; Rider et al., 2018). Therefore, it is possible that poor perceptions of health among TNB young adults and YCSMW may lead to reduced use of healthcare services and result in higher MM.

Using data from a racially diverse longitudinal cohort study of SGM young adults designated female at birth (Whitton et al., 2019), the current study compared rates of MM among TNB young adults and YCSMW and examined correlates of MM among these groups. Using the minority stress and resilience theory and findings from existing literature, we hypothesize that MM will be associated with experiences of increased minority stressors (victimization and internalized stigma), reduced resilience and social support, recent STI testing, prior use of GAH, and negative perceptions of one’s own physical health. Additionally, we predicted that MM will be associated with racial/ethnic and gender identities, such that Black and TNB young adults will report higher MM compared to White and cisgender counterparts respectively. Finally, we explored whether the associations between independent variables (e.g., minority stressors, healthcare experiences etc.) and MM differed between TNB young adults and YCSMW, hypothesizing that these relationships will be stronger for TNB young adults.

## Methods

### Recruitment and Sampling

Data for this study were drawn from FAB400 which is a longitudinal cohort study of SGM youth and young adults designated female at birth in the Chicagoland area (*N* = 488, Whitton et al., 2019). Participants were recruited from the Chicagoland area at SGM community-based venues and through social media advertisements. FAB400 examined the risk and protective factors associated with development, health outcomes and close relationships among young cisgender sexual minority women, young transgender men, and non-binary individuals designated female at birth. All participants provided written informed consent and all procedures were approved by Northwestern University’s Institutional Review Board. The current study utilized data from Visit 6 of FAB400 (90% retention rate, *N* = 439). Data were reviewed for completeness and 29 participants were removed from the final analyses due to missing data on key variables of interest. The analytic sample (*N* = 410) consists of 134 TNB young adults and 276 YCSMW ages 18–33 years.

### Procedures

FAB400 participants received $50 for completing each assessment via a computer assisted self-interview software. Data for Visit 6 of FAB400 were collected between June 2019 and June 2020. Prior to the onset of the COVID-19 pandemic, participants could choose to complete study visits in our offices or remotely via REDCap. After the onset of COVID-19 in March 2020, all remaining data relevant to the current analyses were collected remotely.

### Measures

#### Outcome Variable

##### Medical Mistrust Related to SGM Identity

The Group-based Medical Mistrust scale is a 12-item measure examining distrust of medical professionals and systems among people of color (Thompson et al., 2004). This measure was adapted by our team to assess medical mistrust related to SGM identity and items were reworded from “people of my ethnic group” to “people who share my LGBTQ identity.” For example, items include statements such as “People who share my LGBTQ identity should not confide in doctors and healthcare workers because it will be used against them” and “I have personally been treated poorly or unfairly by doctors or healthcare workers because of my LGBTQ identity.” Items are scored on a 5-point Likert scale with responses ranging from “Strongly disagree” to “Strongly agree.” Responses are averaged to obtain a total mean score, ranging between 1 and 5 (*α* = 0.91). Higher scores indicate greater levels of MM.

#### Demographics

Participants self-reported their date of birth, current gender identity, race/ethnicity current health insurance status, and annual personal income level. Participants were coded as 0 if they indicated a cisgender identity (i.e., “female”) and 1 if they reported any other gender identity (i.e., “male,” “transgender,” “gender non-conforming,” “gender queer,” or “non-binary”). Race/ethnicity responses were coded into four categories (i.e., White, Black, Hispanic/Latinx, and Other), corresponding with the largest population groups in the Chicagoland area. Participants indicating their ethnicity as Hispanic/Latinx were coded as such, regardless of what they indicated for their racial identity. Participants reported whether they currently had health insurance and their annual personal income from a list of options.

#### Predictor Variables

##### Victimization

We measured experiences of SGM-based victimization and harassment over the last 6 months on a 10-item measure (D’Augelli, 1992; D’Augelli et al., 1998). We updated identity-specific language on this measure for the study (e.g., “Have you been verbally insulted (yelled at, criticized), because you are, or were thought to be gay, lesbian, bisexual, or trans?”). This measure scored items on a 5-point Likert scale with responses ranging from “Never” to “More than ten times.” Higher scores indicate more frequent experiences of LGBT victimization. Responses are averaged to obtain a total mean score (*α* = 0.81).

##### Internalized Stigma

Our measure of internalized sexual minority stigma was adapted from previous measures of internalized homophobia (Nungesser, 1983; Ramirez-Valles et al., 2010) and subsequently validated (Puckett et al., 2017). This 8-item measure assesses participant’s self-reported desire to be heterosexual and includes statements such as “Sometimes I think that if I were straight, I would probably be happier,” and “I feel that being [reported sexual identity piped in] is a shortcoming for me.” Items are rated on a 4-point Likert scale with responses ranging from “Strongly Disagree” to “Strongly Agree.” Responses are averaged to obtain a total mean score (*α* = 0.75).

##### Resilience

The 10-item Connor-Davidson Resilience Scale measures participant’s perceived ability to thrive, despite experiences of adversity (Campbell-Sills & Stein, 2007). Items include statements such as “I am able to adapt when changes occur” and “I believe I can achieve my goals, even if there are obstacles.” Items are rated on a 5-point Likert scale with responses ranging from “Not True At All” to “True Nearly All The Time.” Responses are added to obtain a total score (*α* = 0.93).

##### Perceived Social Support

The Multidimensional Scale of Perceived Social Support is a 12-item measure evaluating the quality of social support as reported by participants (Zimet et al., 1988). The measure assesses the quality of participant’s perceived social support from family, friends and a partner and produces three respective subscale scores (*α* = 0.92). Items include statements such as “There is a special person who is around when I am in need,” “I can talk about my problems with my family,” and “My friends really try to help me.” Items are rated on a 7-point Likert scale with responses ranging from “Very Strongly Disagree” to “Very Strongly Agree.” Higher scores indicate greater levels of social support. Responses are averaged to obtain a mean score for each subscale.

##### Recent STI Testing

One question with “Yes” or “No” responses assessed whether participants pursued STI testing in the last 6 months.

##### History of Gender-Affirming Hormone Use

Participants identifying as transgender or non-binary were asked the following question on a 5-point Likert scale: “Have you ever taken hormones as part of hormone therapy (e.g., testosterone)?” Response options included “No, and unsure if would like to in the future,” “No, and would not like to in the future,” “No, but would like to in the future,” “Yes, I have taken hormones in the past but do not currently take hormones,” and “Yes, I currently take hormones.” A binary variable was created from these responses where participants were coded as 0 if they reportedly had never accessed gender-affirming hormones (i.e., response options 1, 2, and 3), and participants were coded as 1 if they reportedly had previously taken or were currently taking gender-affirming hormones (i.e., response options 4 and 5).

##### Self-rated Health

The Patient-Reported Outcomes Measurement Information System (PROMIS) Global Health measure is a 10-item survey examining overall health functioning as perceived by participants (Hays et al., 2009). Items include statements such as “How would you rate your physical health?” and “To what extent are you able to carry out your everyday physical activities such as walking, climbing stairs, carrying groceries, or moving a chair?” Items are scored on a 5-point Likert scale with responses ranging from “Poor” to “Excellent” or “Not at all” to “Completely.” For our analyses, we used the *t*-score generated from the Global Physical Health subscale which includes questions about physical health, physical function, pain, and fatigue (*α* = 0.77).

### Analysis

We first ran bivariate correlational analyses to test the strength of association between our outcome and hypothesized predictor variables. The outcome variable of interest was MM. Correlates of MM included victimization, internalized stigma, resilience, perceived social support from family, friends and a partner, recent STI testing, and self-rated health. We also ran a separate correlation analysis with TNB participants only to test the strength of association between MM and GAH use. Predictor variables correlated with MM at *p* < 0.1 were included in the next stage of analyses.

Next, we ran several multiple regression models to test our hypotheses. Predictor variables were added to the models in three steps. First, we added demographic variables (i.e., race/ethnicity and gender identity) to the regression model. Next, we added all psychosocial variables that were significantly associated with MM in bivariate analyses at *p* < 0.01 to examine their main effects. Finally, we added gender identity to the model as a moderator variable to conduct moderation analyses.

## Results

### Participants

Table 1 outlines participant characteristics and descriptive statistics. The mean age of our sample was 22.4 years (SD = 3.49, range = 18–33 years). Most of the participants identified their gender identity as cisgender woman (*n* = 277, 67.1%), and the largest proportion of the sample identified their race/ethnicity as Black or African American (*n* = 143, 34.6%). The majority of participants reported an annual personal income level as below $20,000 (*n* = 300, 73.7%) and most reported currently having health insurance (*n* = 349, 85.1%).

**Table 1:** Demographic characteristics and descriptive statistics (*N* = 410)

	*n*	*%*
Gender identity		
Transgender or non-binary	134	32.6
Cisgender	276	67.3
Race/ethnicity		
Black/African American	142	34.6
Latinx or Hispanic	101	24.6
White	111	27.1
Other	56	13.7
Personal income level		
Below $20,000	300	73.7
$20,000–$39,999	76	18.5
$40,000–$49,999	14	3.4
$50,000–$59,999	7	1.7
$60,000–$69,999	7	1.7
$70,000–$79,999	2	0.5
Above $80,000	1	0.2
Completed STI Testing in the last 6 months	172	41.95
Currently using or previously on gender-affirming hormones	40	9.8
Currently has health insurance coverage	349	85.1
	*M*	SD
Age (years)	22.3	3.45

*Note*. Missing data from 3 participants on measure of personal income level

### Bivariate Correlations

Table 2 details results from correlational analyses. MM was significantly and positively correlated with victimization (*r* = 0.17, *p* < 0.001) and recent STI testing (*r* = 0.13, *p* < 0.05). Additionally, MM was significantly and negatively correlated with resilience (*r* =  − 0.16, *p* < 0.01), perceived family support (*r* =  − 0.19, *p* < 0.001), and self-rated health (*r* =  − 0.20, *p* < 0.001). Given significant bivariate relationships, these variables were included in the next stage of analyses. The following variables were not significantly correlated with MM at *p* < 0.01: internalized stigma, perceived support from friends and perceived support from one’s partner. Further, among TNB participants, prior use of GAH was not significantly correlated with MM. Therefore, we did not pursue separate analyses with a sub-sample of TNB participants.

**Table 2:** Means, standard deviations, and Pearson’s correlation coefficient with confidence intervals

Variable	*M*	SD	1	2	3	4	5	6	7	8	9
1. Medical Mistrust^a^	2.69	0.71									
2. Victimization^a^	0.07	0.21	.17**								
			[.08, .27]								
3. Internalized Stigma^b^	1.52	0.51	.05	.19**							
			[− .05, .14]	[.10, .28]							
4. Resilience^c^	26.86	7.75	− .16**	− .10*	− .22**						
			[− .26, − .07]	[− .20, − .01]	[− .31, − .13]						
5. Support from Family^d^	4.64	1.60	− .19**	− .03	− .05	.24**					
			[− .29, − .10]	[− .13, .06]	[− .15, .04]	[.15, .33]					
6. Support from Friends^d^	5.58	1.33	− .09	− .08	− .17**	.34**	.43**				
			[− .18, .01]	[− .18, .02]	[− .26, − .07]	[.25, .42]	[.35, .51]				
7. Support from Partner^d^	5.74	1.43	− .07	− .04	− .13**	.23**	.37**	.47**			
			[− .16, .03]	[− .14, .06]	[− .23, − .03]	[.13, .32]	[.28, .45]	[.39, .54]			
8. Recent STI Testing	-	-	.13*	.06	− .05	− .03	− .10*	− .05	− .04		
			[.03, .22]	[− .04, .16]	[− .15, .05]	[− .12, .07]	[− .19, − .00]	[− .15, .05]	[− .13, .06]		
9. Self-rated Health^e^	48.67	7.50	− .20**	− .15**	− .21**	.43**	.19**	.23**	.10*	− .12*	
			[− .29, − .11]	[− .24, − .05]	[− .30, − .12]	[.35, .51]	[.10, .28]	[.13, .32]	[.00, .19]	[− .21, − .02]	
10. History of GAH^+^	-	-	.07	.24**	.07	− .01	.02	− .05	− .01	.04	− .04
			[− .10, .24]	[.07, .39]	[− .10, .23]	[− .17, .16]	[− .15, .18]	[− .21, .12]	[− .18, .16]	[− .13, .21]	[− .21, .13]

*Note. M* and SD are used to represent mean and standard deviation, respectively. Values in square brackets indicate the 95% confidence interval for each correlation

^*^p < 0.05

^**^p < 0.01

^a^Absolute range, 1–5

^b^Absolute range, 1–4

^c^Absolute range, 0–40

^d^Absolute range, 1–7

^e^Absolute range, 0–100

^+^Results in this row derived from a sub-sample of TNB participants only

### Multiple Regression Models

Table 3 details results from three multiple regression models. The first model included demographic variables (race/ethnicity and gender identity) and was significant, accounting for 8.9% of the variance in MM (*R*^2^ = 0.089, *F*(4, 405) = 10.95, *p* < 0.001). Participants with TNB (*β* = 0.46, *p* < 0.001) and Black identities (*β* = 0.22, *p* < 0.05) reported significantly higher MM compared to cisgender and White participants, respectively.

**Table 3:** Multiple linear regression models

	Model 1		Model 2		Model 3	
	*β* (SE)	*p*-value	*β* (SE)	*p*-value	*β* (SE)	*p*-value
*Intercept*	2.44*** (0.07)	< 0.000	3.29*** (0.24)	< 0.000	2.40*** (0.08)	< 0.000
*TNB Identity*	0.47*** (0.07)	< 0.000	0.38*** (0.07)	< 0.000	0.50*** (0.13)	< 0.000
*Race/Ethnicity* —* Black*	0.22* (0.09)	0.01	0.23* (0.09)	0.01	0.33** (0.11)	0.002
*Race/Ethnicity* — *Latinx*	0.07 (0.09)	0.48	0.06 (0.09)	0.51	0.14 (0.12)	0.23
*Race/Ethnicity* — *Other*	− 0.02 (0.11)	0.86	− 0.05 (0.11)	0.62	0.004 (0.14)	0.98
*Victimization*	-	-	0.42** (0.16)	0.007	0.37* (0.18)	0.046
*Resilience*	-	-	− 0.005 (0.005)	0.34	− 0.003 (0.006)	0.58
*Support from Family*	-	-	− 0.053* (0.02)	0.02	− 0.08** (0.03)	0.002
*Recent STI Testing*	-	-	0.004^^^ (0.002)	0.10	0.002 (0.003)	0.55
*Self-rated Health*	-	-	− 0.01* (0.005)	0.046	− 0.009 (0.006)	0.12
*Race/Ethnicity* —* Black x TNB Identity*	-	-	-	-	− 0.30 (0.19)	0.12
*Race/Ethnicity* — *Latinx x TNB Identity*	-	-	-	-	− 0.23 (0.19)	0.23
*Race/Ethnicity* — *Other x TNB Identity*	-	-	-	-	− 0.12 (0.22)	0.60
*Victimization x TNB Identity*	-	-	-	-	0.16 (0.35)	0.65
*Resilience x TNB Identity*	-	-	-	-	− 0.01 (0.01)	0.37
*Support from Family x TNB Identity*	-	-	-	-	0.11* (0.05)	0.03
*Recent STI Testing x TNB Identity*	-	-	-	-	0.004 (0.005)	0.36
*Self-rated Health x TNB Identity*	-	-	-	-	− 0.005 (0.01)	0.66
*Overall* model statistics	*R*^2^ = .089, *F*(4, 405) = 10.95, *p* < 0.000		*R*^2^ = .147, *F*(9, 400) = 8.85, *p* < 0.000		*R*^2^ = .152, *F*(17, 392) = 5.32, *p* < 0.000	

^***^p < 0.000, **p < 0.001, *p < 0.05, ^*p* < 0.1

The second model comprised of demographic variables and predictor variables which were previously noted to be significantly correlated with MM in bivariate analyses. These variables included victimization, resilience, perceived support from family, recent STI testing and self-rated health. This model was overall significant, accounting for 14.7% of the variance in MM (*R*^2^ = 0.147, *F*(9, 400) = 8.85, *p* < 0.001). Like the previous model, TNB and Black identities remained significantly associated with higher MM. Among psychosocial independent variables, victimization (*β* = 0.42, *p* < 0.01) was significantly associated with MM, such that MM increased with higher rates of victimization. Additionally, family support (*β* =  − 0.05, *p* < 0.05) was associated with MM, such that increases in perceived family support were associated with decreases in MM. Further, self-rated health was significantly associated with MM (*β* =  − 0.01, *p* < 0.05), such that MM increased as perceived health worsened. Finally, recent STI testing and resilience were not associated with MM in this model, after controlling for all other variables.

**Fig. 1:**
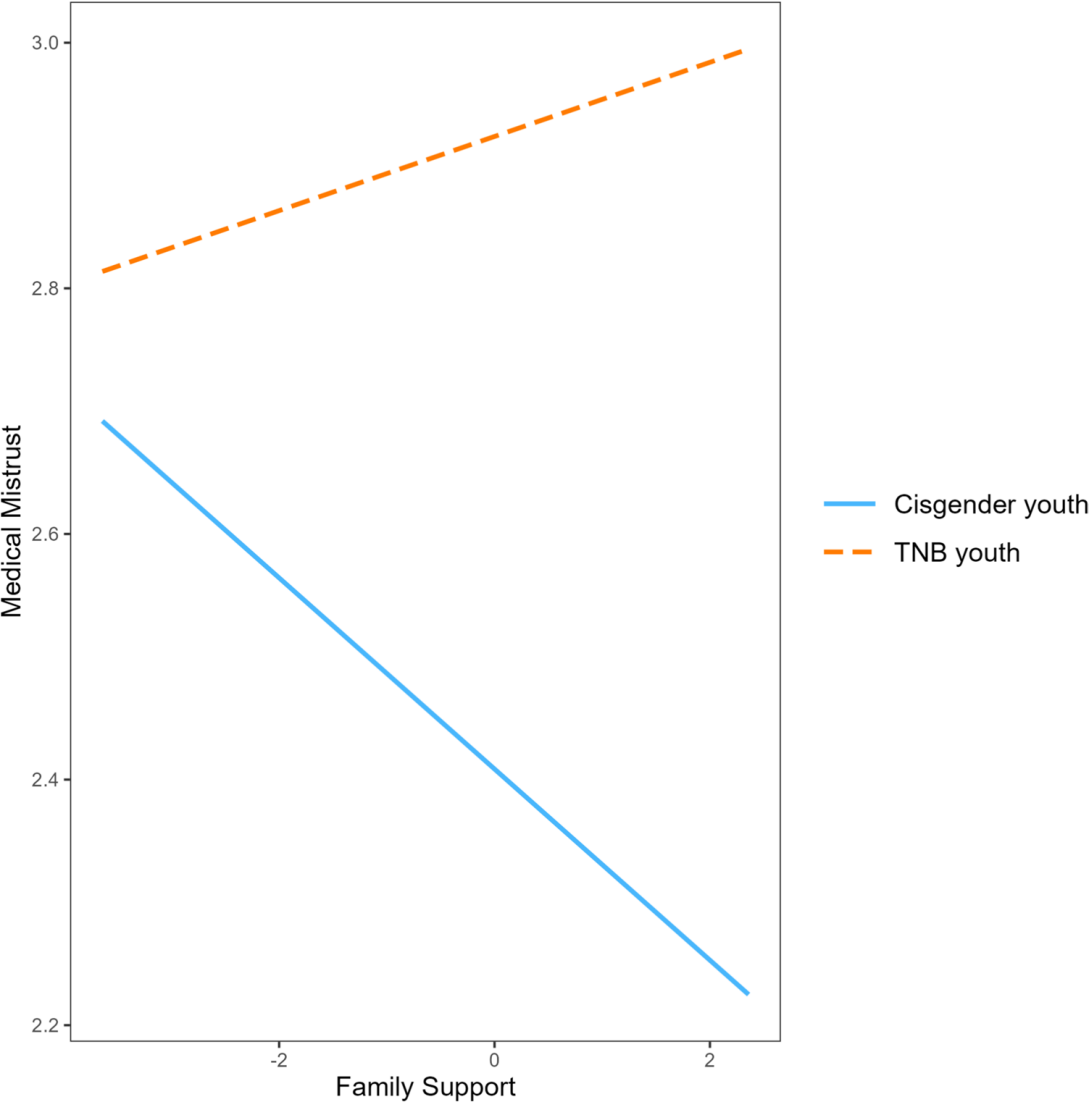
Moderating effect of gender identity on the relationship between medical mistrust and family support

The third model comprised of demographic, predictor, and moderating variables and was overall significant, accounting for 15.2% of the variance in MM (*R*^2^ = 0.152, *F*(17, 392) = 5.32, *p* < 0.001). All previously described demographic and main effects remained significant in this model, except self-rated health which was not significantly associated with MM in this model. Additionally, gender identity moderated the relationship between perceived family support and MM (*β* = 0.11, *p* < 0.05), such that MM decreased as perceived family support increased among cisgender participants (Fig. 1). However, this effect was not observed among TNB participants. Gender identity did not moderate the relationships between MM and any other predictor variables.

Finally, we compared the fit of our three models using an analysis of variance. We observed that the second model (i.e., main effects model) which included demographic and predictor variables best described our data. The moderation model added eight predictors to the model, however the amount of variance explained by the moderation model was similar to the variance explained by the main effects model. Despite adding additional predictors, the moderation model did not explain significantly more variance relative to the second model. Therefore, we concluded that the main effects model best described our data.

## Discussion

Our study compared group differences in rates of MM among a sample of racially diverse SGM young adults designated female at birth. We found that TNB and Black young adults in our sample reported greater MM compared to young cisgender sexual minority women and White young adults, respectively. We also examined correlates of MM among these groups in line with the minority stress and resilience theory and findings from existing MM literature. In multivariate models, we observed that higher rates of victimization and poorer perceptions of one’s own health were associated with increased MM. Additionally, higher family support was associated with reduced MM. Finally, moderation analyses indicated that there were differences in the strength of the association between perceived family support and MM for TNB young adults and YCSMW, such that the promotive effective of family support on reduced MM was only present among YCSMW.

Given high rates of MM reported by young adults in our study, our findings underscore the need to understand the development of MM among SGM populations. High rates of MM during late adolescence and young adulthood may translate to even greater MM during adulthood, which may exacerbate current and future health problems. In our sample, we also observed some differences in MM between groups of SGM young adults. First, Black individuals indicated higher MM compared to White peers. This finding is consistent with existing literature documenting higher MM among Black populations due to structural racism and histories of medical violence (Benkert et al., 2019; Thompson et al., 2004). Additionally, higher levels of MM have also been noted among Black SGM populations, mostly in the context of HIV and PrEP and related care, and among Black cisgender women. Our findings extend the current literature by documenting the same patterns of high MM among Black TNB young adults designated female at birth and YCSMW. Future interventions targeting MM must specifically address developmental and intersectional influences of structural stigma, especially among Black SGM young adults.

Second, we observed higher rates of MM in TNB young adults, relative to YCSMW. To our knowledge, this is the first study to examine rates of MM among TNB young adults designated female at birth and compare rates of MM among this group to cisgender counterparts. Prior studies of MM among TNB populations have predominantly focused on transfeminine individuals in the context of HIV-related care. Our study expands existing literature by investigating MM in an underrepresented sub-population (i.e., TNB young adults designated female at birth) and in the context of general (i.e., non-HIV related) healthcare. Our findings are consistent with previous studies which have noted high levels of MM among transfeminine individuals (e.g., D’Avanzo et al., 2019; Sevelius et al., 2016).

Regarding victimization, there is substantial evidence documenting experiences of stigma and discrimination encountered by SGM young adults within the healthcare system (Chong et al., 2021; Goldenberg et al., 2019; Kearns et al., 2021). At the same time, research studies examining clinician perspectives have noted that providers working with SGM young adults frequently struggle with personal discomfort, fear of offending patients, and lack of training in SGM-affirming practices (Forsberg & Eliason, 2022; Rider et al., 2019). In fact, a review of physician training program curricula found that medical students on average only receive 1–2 hours of education on SGM health related topics (Utamsingh et al., 2017). Taken together, SGM young adults may experience suboptimal care from both overtly biased and well-intentioned providers, which likely increases their MM. Therefore, SGM-inclusive education programs and supplementary trainings may enable clinicians to provide affirming healthcare to SGM young adults, develop stronger patient-provider relationships, and decrease MM.

It is important to note that our measure of victimization did not specifically query about SGM young adults’ experiences within the healthcare system, so participants likely did not reflect on their healthcare experiences when answering these questions. Given our findings, it stands to reason that general experiences of victimization, albeit outside of medical settings, can also translate to distrust of the healthcare system. One possible explanation for this finding is offered by the institutional performance theory (Norris, 2007) which states that individuals may evaluate the effectiveness of an institution based on their confidence in other large-scale systems (e.g., insurance, housing, and education systems). There is evidence indicating that SGM populations are more likely to be uninsured (dickey et al., 2016; Gonzales & Henning-Smith, 2017), face houselessness (Corliss et al., 2011; Rice et al., 2012) and peer victimization in schools (Reisner et al., 2015a, 2015b; Tucker et al., 2016). Such negative experiences encountered within other systems may lead to a distrust of the healthcare system. This finding also supports the “group-based” conceptualization of MM which asserts that individuals (usually belonging to marginalized social groups) may believe that the trustee, usually an individual belonging to a different social group *or* a larger system, will work against their best interests (Benkert et al., 2019; Thompson et al., 2004). In this way, MM goes beyond interpersonal trust in individual providers and implicates larger healthcare systems.

Regarding social support, there is growing evidence to suggest that family support buffers against the effects of minority stressors and builds resilience among SGM young adults (Olson et al., 2016; Puckett et al., 2019). Our findings add to this literature and demonstrate that higher perceived family support is associated with less MM. Given that our study examined family support as a main effect (rather than a moderator of the link between MM and other health outcomes), we cannot say whether family support indeed has a “stress-buffering” effect against MM. Future studies should examine whether family support may have a protective effect on the relationship between minority stressors and MM. Regardless, the main effect observed in these analyses indicates that perceiving support from one’s family may promote more trust in the medical system. It is possible that young adults with greater family support may feel more secure and trustworthy of others around them. On the contrary, young adults who do not feel supported by their family may be cautious of others, including the medical system. As observed by our moderation analyses, such effects may be more pronounced for cisgender compared to TNB young adults. Therefore, perceiving support from one’s family may increase trust in the healthcare system and act as a health promoting factor for cisgender young adults. However, this finding should be interpreted with caution since we concluded that the main effects model best described our data. Future studies should investigate the relationships between MM, family support and gender identity.

The minority stress and resilience theory posits that social support and resilience may serve as protective factors, buffering against minority stressors to promote health outcomes. However, in our study, we did not find significant associations between MM, the trait of resilience, and support from friends or a significant other. It is likely that SGM young adults’ friends and significant others are also part of the SGM community and may share similar levels of MM. Further, given that friends and significant others are likely of similar age to our participants (i.e., late adolescents and emerging adults), so they may not yet have sufficient experience with the healthcare system to help SGM young adults navigate the medical system. Given this evidence for family support over other types of social support and protective factors, future interventions should target increasing family support to address MM and associated health disparities among SGM young adults.

Our study results indicated that poor self-perceptions of physical health were associated with greater levels of MM. This finding aligns with existing literature documenting this relationship in the general population (Armstrong et al., 2006). Given that self-rated health is a valid measure of health status (Miilunpalo et al., 1997), individuals reporting poorer self-rated health may have health conditions requiring frequent interaction with the healthcare system which may exacerbate MM. Conversely, individuals with greater MM may avoid engaging with the healthcare system in ways that may improve their health. Although sexual minority women experience several physical health disparities (Simoni et al., 2017) and may come into contact with the medical system more frequently, there is mixed evidence in the literature around whether TNB individuals experience greater physical health concerns compared to peers (Levit et al., 2021; Rider et al., 2018; Zwickl et al., 2023). Therefore, it is likely that there are additional mechanisms underlying this association. For example, it is possible that individuals with greater MM are slow to adopt the most up to date health information and treatment, which could negatively affect one’s physical health. Future studies should investigate the relationship between health problems and self-rated health among SGM young adults.

In contrast, we observed that recent STI testing was not associated with MM for SGM young adults. Previous literature has examined the negative influence of MM on uptake of HIV prevention strategies among sexual minority men and transgender women (Arrington-Sanders et al., 2020); however, this topic has been understudied among SGM young adults designated female at birth. In our study, around 42% of participants reported completing STI testing, other than for HIV, in the last 6 months. STI testing services are unique as they can be provided at a community health center, usually located outside a traditional hospital setting, and can be completed by a trained paraprofessional. Additionally, these services may be offered through public programs for free or low cost. Further, STI testing venues may be more sex-positive and affirming of SGM individuals compared to traditional medical services. Therefore, it is surprising that having recently had STI testing was not associated with *decreased* MM. One possible explanation is that our measure of STI testing was too broad. We assessed recent STI testing as a dichotomous (i.e., yes or no) variable where participants indicated whether they had received STI testing, in the last 6 months. We did not assess the *quality* of these experiences (e.g., whether they were affirming or positive medical experiences) or where they occurred. Future studies should examine whether perceived quality of STI testing experiences increases MM towards other, more general, medical services.

Results from our moderation analyses indicated that victimization, the trait of resilience, and recent STI testing did not impact MM among TNB young adults at a greater rate compared to YCSMW. TNB young adults reporting higher levels of MM compared to cisgender counterparts likely stems from higher reported levels of victimization among these individuals. It is also possible that MM among TNB young adults may be associated with unique, higher-level factors at the neighborhood, cultural or ecological levels which our study did not examine. For example, in the current sociopolitical climate of the United States, there have been increased restrictions placed on gender-affirming policies including social affirmation of TNB individuals, participation in sports and healthcare provision. At the federal-level, several executive orders prohibiting gender-affirming healthcare access for TNB individuals have been announced in 2025 (Wolf, 2025). At the state-level, 137 healthcare related bills in 2023, 112 bills in 2024, and 132 bills in 2025 thus far have been introduced restricting gender-affirming care access for TNB individuals (American Civil Liberties Union, 2025). Such policies may further distrust of the healthcare system and exacerbate existing health disparities for TNB young adults. This may occur even in states where these policies are not being enacted (e.g., the Chicagoland area, where data for this study was collected and where protective laws at the state-level have been enforced). It is important to note that our data were collected during 2019–2020, before the current wave of policies restricting gender affirmation. Therefore, it is highly likely that current rates of MM among TNB individuals are higher than what our data indicate. Future research should examine these relationships and factors buffering against the negative impact of such policies. Additionally, continued advocacy efforts against the passing of such legislature may have downstream effects of improving MM among TNB young adults.

### Strengths and Limitations

Our study adds to the literature on MM among understudied populations (i.e., TNB and cisgender sexual minority young adults designated female at birth). Despite the strengths, findings should be considered in the context of our limitations. Due to the cross-sectional nature of the study, we are unable to predict the direction of effects. Therefore, it is possible that MM may cause, rather than be a consequence of, the correlates examined in our study (e.g., poor self-rated health). Future studies should utilize longitudinal data to understand the development of MM over time. Additionally, our measure of STI testing was broad and did not assess the quality of the experience (i.e., whether these were affirming or positive medical experiences). Further, we were unable to disaggregate participants who identified with a racial/ethnic group other than White, Black, or Hispanic/Latinx. This was done because the cell sizes within these categories were small, and we could not reliably measure their associations with MM. We decided to break out race/ethnicity categories to align with the three largest racial/ethnic groups in the Chicagoland area. Therefore, findings from our study may not generalize to young adults belonging to other racial/ethnic groups, especially those living outside the Chicagoland area. Lastly, our study utilized a community sample of SGM young adults designated female at birth and may not be representative of the national SGM young adult population.

## Conclusion

Our study examined correlates of MM among a community sample of SGM young adults designated female at birth. We observed that TNB and Black young adults reported higher MM compared to cisgender and White counterparts. Additionally, we noted that higher victimization, lower family support and poor self-rated health were associated with increased MM among our sample. Finally, gender identity moderated the relationship between family support and MM, such that this effect was stronger for cisgender young adults. However, this finding must be interpreted with caution. Longitudinal research is needed to examine how these factors predict MM over time. Interventions to reduce MM should consider intersectional stigma, clinician competencies around working with SGM young adults and increasing family support. Additionally, structural approaches involving policy changes improving access to healthcare services are needed to improve MM among SGM young adults.

## Data Availability

Data used for this manuscript are not currently publicly available but can be made available upon request.

## Code Availability

Available upon request.
